# The association between the expression of nuclear Yes-associated protein 1 (YAP1) and p53 protein expression profile in breast cancer patients

**DOI:** 10.1371/journal.pone.0250986

**Published:** 2021-05-10

**Authors:** Yoon Jin Cha, Dooreh Kim, Soong June Bae, Sung Gwe Ahn, Joon Jeong, Min Kyung Cho, Pill Sun Paik, Tae-Kyung Yoo, Woo-Chan Park, Chang Ik Yoon

**Affiliations:** 1 Department of Pathology, Gangnam Severance Hospital, Yonsei University College of Medicine, Seoul, Korea; 2 Department of Surgery, Gangnam Severance Hospital, Yonsei University College of Medicine, Seoul, Korea; 3 Division of Breast Surgery, Department of Surgery, Seoul St Mary’s Hospital, College of Medicine, The Catholic University of Seoul, Seoul, Korea; Fondazione IRCCS Istituto Nazionale dei Tumori, ITALY

## Abstract

**Background:**

Yes-associated protein 1 (YAP1) is a key effector molecule regulated by the Hippo pathway and described as a poor prognostic factor in breast cancer. Tumor protein 53 (TP53) mutation is well known as a biomarker related to poor survival outcomes. So far clinical characteristics and survival outcome according to YAP1 and TP53 mutation have been poorly identified in breast cancer.

**Patients and methods:**

Retrospectively, 533 breast tumor tissues were collected at the Seoul St Mary’s hospital and Gangnam Severance Hospital from 1992 to 2017. Immunohistochemistry with YAP1 and p53 specific antibodies were performed, and the clinical data were analyzed.

**Results:**

Mutant p53 pattern was associated with aggressive tumor features and advanced anatomical stage. Inferior overall survival (OS) and recurrence free survival (RFS) were related with mutant p53 pattern cases with low nuclear YAP1 expression (*P* = 0.0009 and *P* = 0.0011, respectively). Multivariate analysis showed that mutant p53 pattern was an independent prognostic marker for OS [hazard ratios (HR): 2.938, 95% confidence intervals (CIs): 1.028–8.395, P = 0.044] and RFS (HR: 1.842, 95% CIs: 1.026–3.304). However, in cases with high nuclear YAP1 expression, there were no significantly difference in OS and RFS according to p53 staining pattern.

**Conclusion:**

We found that mutant p53 pattern is a poor prognostic biomarker in breast tumor with low nuclear YAP1 expression. Our findings suggest that interaction between nuclear YAP1 and p53 expression pattern impact survival outcomes.

## 1. Background

Yes-associated protein 1 (YAP1) is a key downstream effector molecule of the Hippo pathway. The Hippo pathway is a central regulator of organ size and tissue homeostasis. In addition, YAP1 is involved in the control of cell proliferation, stem cell maintenance, metastasis, apoptosis, and tumor differentiation [1–6]. Activated YAP1 is generally considered to be an oncogene in diverse solid cancers [7–9], but there is still controversy regarding the role of YAP1 of breast cancer [10, 11]. The p53 protein is functionally inactivated in most of the human malignancies due to both alterations in its regulatory pathways and mutations that directly affect the tumor protein 53 (TP53) gene [12, 13]. Moreover, mutations of the TP53 is associated with poor survival outcome in solid cancers including breast cancer [14, 15]. Critical for function of tumorigenesis is the ability of mutant p53 protein to be engaged in aberrant molecular interactions that lead to dramatic alterations in gene expression. In several studies, mutant TP53 has been shown to interact with several transcription factors such as nuclear transcription factor Y, sterol regulatory-element binding proteins, specificity protein 1, and vitamin D receptor [16–19]. YAP1 physically interacts with mutant p53 proteins in breast cancer cells and mutant TP53 enhanced pro-proliferative transcriptional activity such as cyclin A, cyclin B, and cyclin dependent kinase 1 genes [20].

However, the clinical characteristics and survival outcomes according to nuclear YAP1 and p53 co-expression in breast cancer have been poorly explored. The aim of this study was to explore clinical characteristics and survival outcomes of patients with breast cancer according to the expression level of nuclear YAP1 and p53 protein expression pattern.

## 2. Material and methods

### 2.1. Patients

We retrospectively collected tumor tissues from patients undergoing primary curative surgery for breast cancer at the Gangnam Severance Hospital in Seoul, Korea from February 1992 to April 2017 and at the Seoul St Mary’s hospital in Seoul, Korea from March 2012 to October 2017. Enrolled patients were underwent curative surgery with histologically confirmed invasive breast cancer (stage Ⅰ to Ⅲ). All patient treatments were performed according to standard protocols. Clinical data including age at surgery, tumor size, lymph node status, histological grade (HG), status of estrogen receptor (ER), status of progesterone receptor (PR), status of human epidermal growth factor receptor-2 (HER2), lympho-vascular invasion (LVI), treatment modalities, recurrence, and death. Tumor HG was determined by applying the modified Scarff-Bloom-Richardson grading system. The median age in this study was 50 years (range, 14–86 years). All procedures performed in studies involving human participants were in accordance with the ethical standards of the institutional and/or national research committee and with the 1964 Helsinki Declaration and its later amendments or comparable ethical standards. The study protocol was approved by the institutional review board (IRB) of the Gangnam Severance Hospital (local IRB No. 3-2019-0188) and St Mary’s hospital (Local IRB number: KC17TNSI0414). The need for informed consent was waived under the approval of the IRB due to the retrospective design. All data was completely anonymized prior to analysis. Patients’ medical records were assessed from 1995 to 2015.

### 2.2. Immunohistochemistry staining and interpretation

As previously described [21], 3-μm thick tissue sections were cut from formalin-fixed paraffin-embedded (FFPE) tissue microarray (TMA) blocks. After deparaffinization and rehydration with xylene and alcohol graded solutions, respectively, immunohistochemistry (IHC) was performed by using a Ventana Discovery XT Automated Slide Stainer (Ventana Medical System, Tucson, AZ, USA). Cell Conditioning 1 buffer (citrate buffer, pH 6.0; Ventana Medical System) was used for antigen retrieval. The slices were incubated with primary antibody against p53 (1:300, clone DO-7; Novocastra, Leica Biosystems, Newcastle Upon Tyne, UK), YAP1 (1:200, clone 63.7, Santa Cruz Biotechnology, Dallas, Texas, USA), ER (ER; 1:150, clone 6F11; Novocastra), PR (PR; 1:100; clone 16; Novocastra), HER2 (1:1500, DAKO, Glostrup, Denmark, clone polyclonal). The appropriate positive and negative controls were included.

#### 2.2.1 Molecular subtyping

Nuclear staining values of 1% or higher were considered indicative of ER and PR positivity [22]. HER2 staining was interpreted based on the 2018 American Society of Clinical Oncology/College of American Pathologists guidelines [23]. Only samples with strong and circumferential membranous HER2 immunoreactivity (3+) were considered as positive, while those with 0 and 1+ HER2 staining were regarded as negative. Cases with equivocal HER2 expression (2+) were further evaluated for HER-2 gene amplification by silver in situ hybridization (SISH). Breast cancer subcategorization was based on the results of IHC staining for ER, PR, HER2, as well as the SISH results for HER2. The specimens were categorized as follows: i) Luminal/HER2-negative (ER- and/or PR-positive and HER2-negative); ii) HER2-positive (HER2-positive regardless of ER and PR status); iii) triple negative breast cancer (TNBC; ER-, PR-, and HER2-negative).

#### 2.2.2. Interpretation of YAP1 and p53 immunohistochemistry

**Fig 1:**
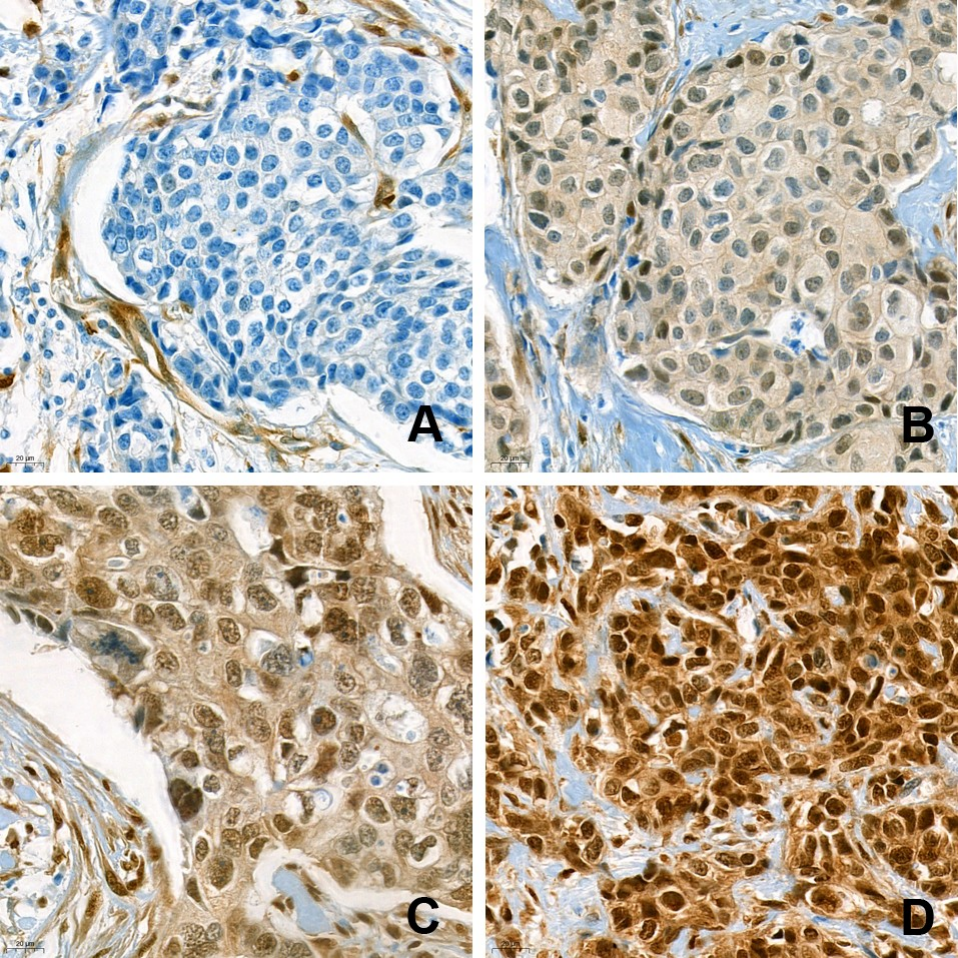
Immunohistochemical analysis of nuclear YAP1 expression. IHC analysis was evaluated in high-power fields (400x magnification) by an experienced pathologist. The sample were classified as negative (a), 1+ (b), 2+ (c), and 3+ (d), based on the intensity of YAP1 nuclear staining.

As previously described [24], tumors with negative and weak (1+) nuclear YAP1 staining were considered low YAP1 tumors, while high YAP1 tumors were defined as moderate (2+) and strong (3+) nuclear YAP1 expression (Fig 1).

**Fig 2:**
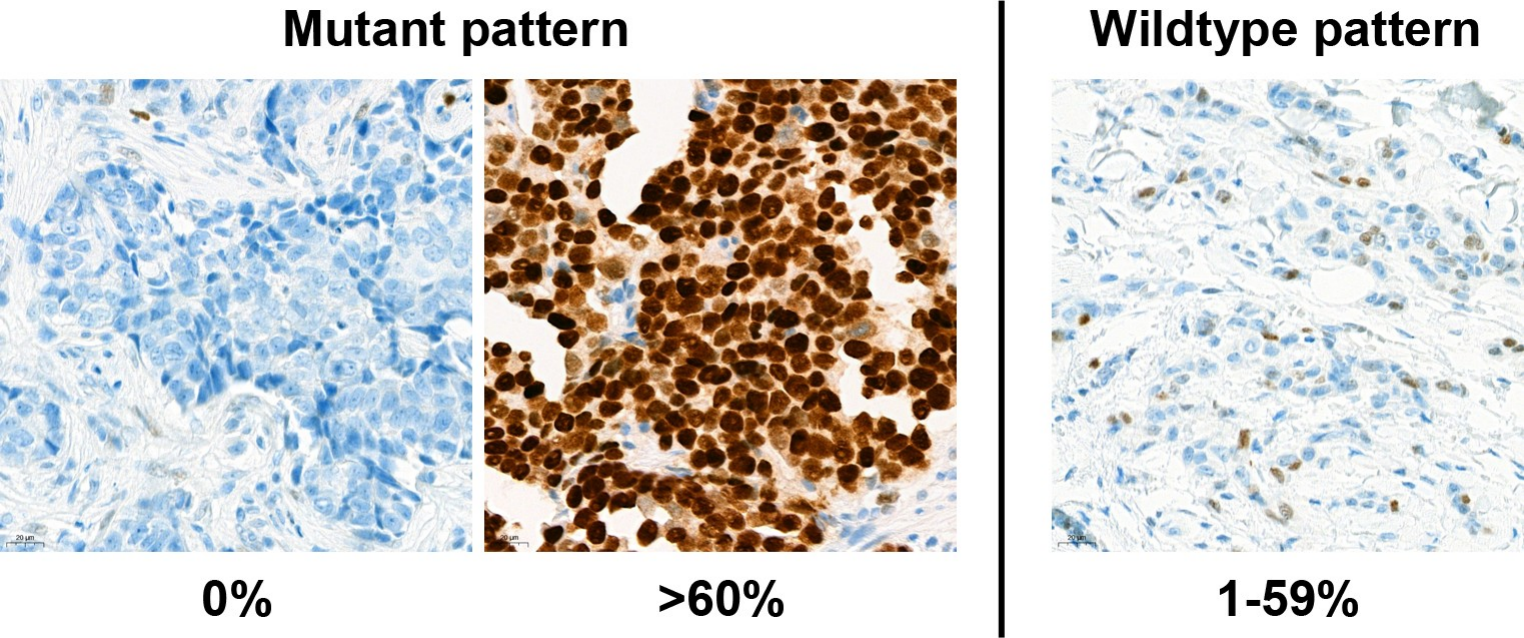
Expression pattern of p53. (Left) Mutant p53 pattern was defined when tumor completely loss of p53 expression or more than 60% of tumor cells had nulear p53 expression. (Right) Except for mutant p53 pattern, various random expression ranged from 1% to 59% of p53 nuclear expression was considered as wild-type expression pattern.

The p53 IHC was interpreted as two mutant patterns and wild type pattern [25, 26] (Fig 2):

1. nonsense mutation pattern: completely absence of expression (0%)
2. missense mutation pattern: diffuse and strong expression (>60% of tumor cell nuclei)
3. wild type pattern: focal and weak expression

### 2.3. Statistical analysis

Overall survival (OS) was defined as the period from the date of curative primary surgery to the end of follow-up or death due to any cause. Recurrence free survival (RFS) was defined as the period from the date of primary surgery to the date of any recurrence (loco-regional and/or distant metastasis) of breast cancer, death due to any cause or the last follow-up. The data of patients who did not exhibit relevant events were censored at the end of follow-up.

Continuous variables between two groups were compared using the Student’s *t*-test or the Mann-Whitney test. Categorical variables were compared by using the *Chi*-square test or the Fisher’s exact test. Survival curves were obtained by the Kaplan-Meier method and two-group comparisons were performed by log-rank test. The uni- and multivariate Cox proportional hazard models were used to identify factors associated with survival outcome (OS and RFS). The variables used in the multivariate analysis were those that showed statistical significance in the univariate analysis.

Statistical analysis was performed by using SPSS version 24 (SPSS: Chicago, IL, USA) software. Statistical significance was defined as a *p*-value less than 0.05, and a 95% confidence intervals (CIs) not including 1.

## 3. Results

### 3.1. Impact of p53 mutation on the baseline characteristics of patients with nuclear YAP high/low breast cancer

A total of 553 breast cancer patients at Gangnam Severance Hospital and Seoul St Mary’s Hospital were included in this study. Low and high nuclear YAP1 expression was found in the tumors of 445 (80.5%) and 108 (15.5%) patients, respectively. Among 553 cases, 411 (74.3%) had wild-type p53 pattern, 142 (25.7%) had mutant p53 pattern, respectively. The clinical characteristics between patients in these two groups according to nuclear YAP1 expression were compared in Table 1. Mutant p53 pattern with low nuclear YAP1 expression was associated with aggressive tumor features, including ER negativity, PR negativity, high HG, aggressive subtype (TNBC), larger tumor size, receipt of chemotherapy, radiotherapy, and/or endocrine therapy. Mutant p53 pattern with high nuclear YAP1 expression also was related to aggressive feature, including ER negativity, PR negativity, high HG, LVI, receipt of chemotherapy, and endocrine therapy.

**Table 1:** Clinical characteristics in relation to nuclear YAP1 expression and p53 expression pattern.

	Nuclear YAP1 Low			Nuclear YAP1 High		
	WT p53 pattern, n = 329, (%)	Mutant p53 pattern, n = 116, (%)	*P* value	WT p53 pattern, n = 82, (%)	Mutant p53 pattern, n = 26, (%)	*P* value
**Age (year, mean±SD)**	51.42±11.12	51.90±11.51	0.692	49.79±9.46	50.15±9.28	0.865
**ER**			<0.001			0.009
**Positive**	210 (63.8)	23 (19.8)		32 (39.0)	3 (11.5)	
**Negative**	119 (36.2)	93 (80.2)		50 (61.0)	23 (88.5)	
**PR**			<0.001			0.046
**Positive**	181 (55.0)	16 (13.8)		26 (31.7)	3 (11.5)	
**Negative**	148 (45.0)	100 (86.2)		56 (68.3)	23 (88.5)	
**HER2**^a^			0.147			0.446
**Positive**	34 (10.3)	18 (15.5)		9 (11.0)	1 (3.8)	
**Negative**	291 (88.4)	98 (84.5)		73 (89.0)	25 (96.2)	
**Missing**	4 (1.2)	0		0	0	
**HG**^**a**^			<0.001			<0.001
**I, II**	210 (63.8)	27 (23.3)		50 (61.0)	4 (15.4)	
**III**	114 (34.7)	89 (76.7)		31 (37.8)	21 (80.8)	
**Missing**	5 (1.5)	0		1 (1.2)	1 (3.8)	
**Subtype**^a^			<0.001			0.010
**Luminal/HER2(-)**	200 (60.8)	18 (15.5)		31 (37.8)	3 (11.5)	
**HER2 (+)**	32 (9.7)	18 (15.5)		9 (11.0)	1 (3.8)	
**TNBC**	93 (28.3)	80 (69.0)		42 (51.2)	22 (84.6)	
**Missing**	4 (1.2)	0		0	0	
**Tumor size**			0.010			0.162
**≤2 cm**	179 (45.4)	47 (40.5)		38 (46.3)	8 (30.8)	
**>2 cm**	150 (45.6)	69 (59.5)		44 (53.7)	18 (69.2)	
**Lymph node metastasis**^a^			0.593			0.996
**Negative**	221 (67.2)	75 (64.7)		53 (64.6)	17 (65.4)	
**Positive**	107 (32.5)	41 (35.3)		28 (34.1)	9 (34.6)	
**Missing**	1 (0.3)	0		1 (1.2)	0	
**LVI**^a^			0.293			0.004
**Negative**	245 (74.5)	80 (69.0)		59 (72.0)	11 (42.3)	
**Positive**	73 (22.2)	31 (26.7)		18 (22.0)	13 (50)	
**Missing**	11 (3.3)	5 (4.3)		5 (6.1)	2 (7.7)	
**Chemotherapy** ^a^			<0.001			0.006
**Done**	187 (57.4)	96 (82.8)		53 (64.6)	24 (92.3)	
**Not done**	139 (42.6)	19 (16.4)		29 (35.4)	2 (7.7)	
**Missing**	3 (0.9)	1 (0.9)		0	0	
**Radiotherapy** ^a^			0.010			0.091
**Done**	186 (56.5)	82 (70.7)		38 (46.3)	17 (65.4)	
**Not done**	140 (42.6)	34 (29.3)		44 (53.7)	9 (34.6)	
**Missing**	3 (0.9)	0		0	0	
**Endocrine therapy**			<0.001			0.005
**Done**	227 (69.0)	24 (20.7)		34 (41.5)	3 (11.5)	
**Not done**	100 (30.4)	92 (79.3)		48 (58.5)	23 (88.5)	
**Missing**	2 (0.6)	0		0	0	

WT, wild-type; SD, standard deviation; ER, estrogen receptor; PR, progesterone receptor; HER-2, human epidermal growth factor receptor-2; HG, histological grade; TNBC, triple negative breast cancer; LVI, lymph-vascular invasion.

^a^Percentages calculated without missing values.

Among 553 patients, 275 patients underwent p53 sequencing and IHC assay at the same time. In cases with wild-type p53 pattern IHC (n = 209), there was no mutation by p53 sequencing (S1 Table). In cases with mutant p53 pattern IHC (n = 66), 54 cases (81.8%) showed TP53 mutation by sequencing analysis (including missense, non-sense, and frameshift mutation etc.). Among remaining twelve, three had TP53 mutations of uncertain significance and nine had wild-type TP53.

### 3.2. Prognostic significance of nuclear YAP1 expression

At a median follow-up time of 59 months (range, 0–325 months), 74 experienced recurrences. Among them, 49 had distant metastasis and 31 had loco-regional recurrences (including duplication). There were a total of 24 deaths.

**Fig 3:**
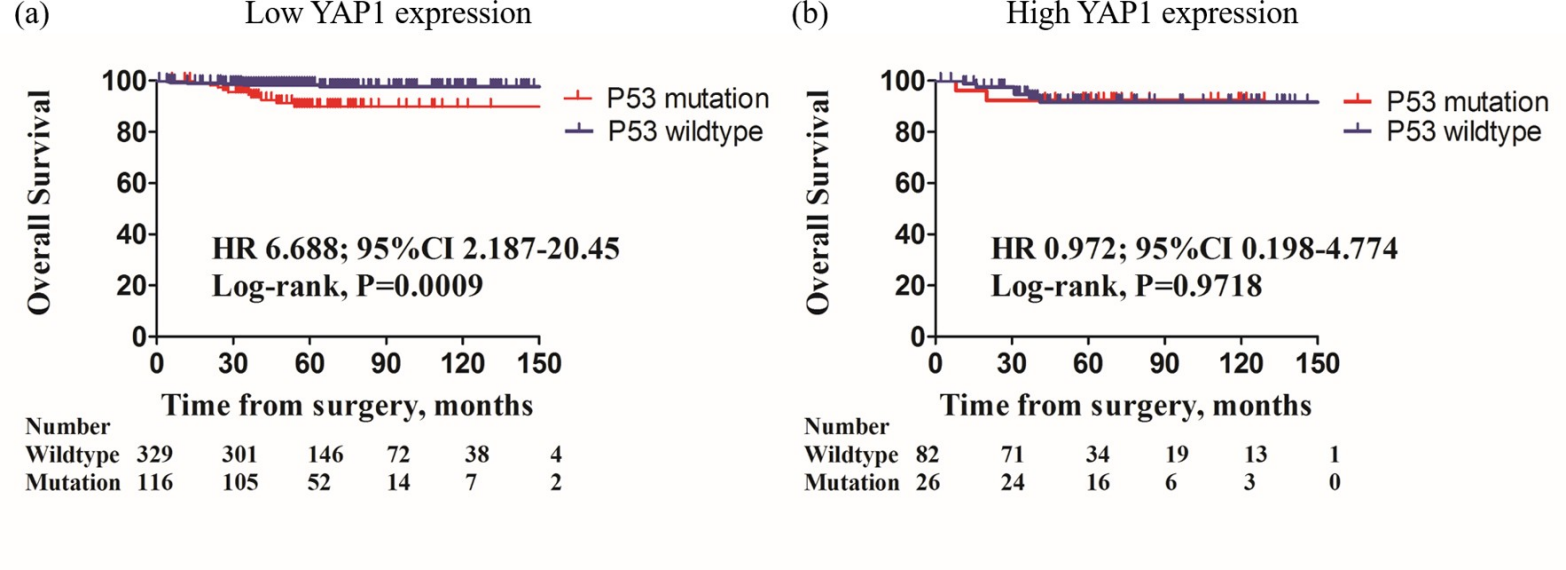
Kaplan-Meier survival curves of OS in according to presence of p53 mutation with low/high nuclear YAP1 expression. In patients with low nuclear YAP1 expression, p53 mutation pattern exhibited poor OS than p53 wild-type pattern (a, HR 6.688, 95% CIs 2.187–20.45, *P* = 0.0009, log-rank test). However, in patients with high nuclear YAP1 expression, p53 mutation pattern was not significantly different OS than p53 wild-type (b, HR 0.972, 95% CIs 0.198–4.774; *P =* 0.9718, log-rank test).

**Fig 4:**
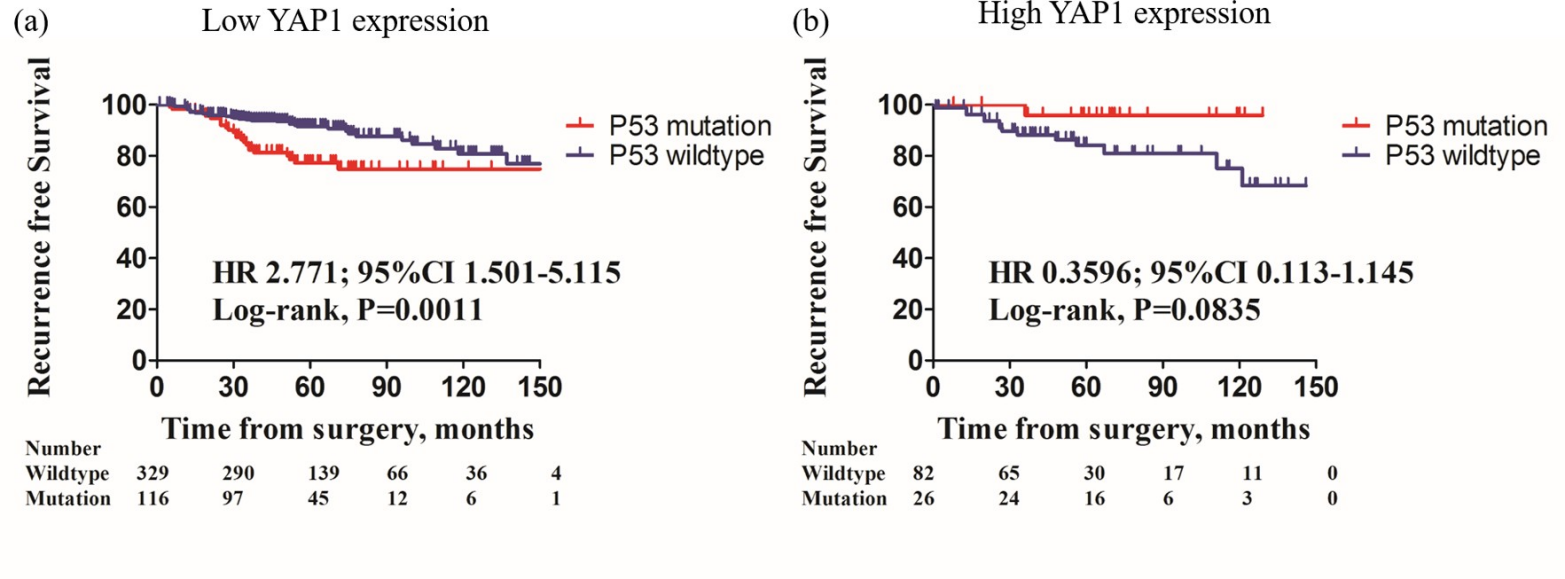
Kaplan-Meier survival curves of RFS in relation to presence of p53 mutation with low/high nuclear YAP1 expression. In patients with low nuclear YAP1 expression, p53 mutation pattern exhibited inferior RFS than p53 wild-type pattern (a, HR 2.771, 95% CIs 1.501–5.115, *P* = 0.0011, log-rank test). However, in patients with high nuclear YAP1 expression, p53 mutation pattern was not significantly different OS than p53 wild-type pattern (b, HR 0.3596, 95% CIs 0.113–1.145; *P =* 0.0835, log-rank test).

Among the low YAP1 tumors, mutant p53 pattern showed a significantly decreased OS compared to those with wild-type p53 patterns [Fig 3A; hazard ratios (HR) 6.688; 95% confidence intervals (CIs) 2.187–20.45, *P* = 0.0009]. Tumors with mutant p53 patterns were also had a significantly poorer RFS compared to those with wild-type p53 pattern (Fig 4A; HR 2.771; 95% CIs 1.501–5.115, *P* = 0.0011).

However, among the high YAP1 tumors, OS (Fig 3B; HR 0.972; 95% CIs 0.198–4.774, *P* = 0.9718) and RFS (Fig 4B; HR 0.3596; 95% CIs 0.113–1.145, *P* = 0.0835) were not differed between mutant and wild-type p53 pattern.

In the univariate Cox proportional hazard model, ER, PR, HG, LVI, mutant p53 pattern, receipt of radiotherapy, and receipt of endocrine therapy were significantly associated with an inferior OS in low YAP1 tumors (Table 2; HR 4.751, 95% CIs 1.726–13.073; *P* = 0.003). After adjustment of these factors, the multivariate Cox proportional hazard model revealed that mutant p53 pattern was a significantly independent prognostic factor for OS in low YAP1 tumors (Table 2; HR 2.938, 95% CIs 1.028–3.395, *P* = 0.044).

**Table 2:** Uni- and multivariate analysis for overall survival in patients with low nuclear YAP1 expression tumors.

	Univariate analysis		Multivariate analysis	
	HR (95% CIs)	*P* value	HR (95% CIs)	*P* value
**Age (continuous)**	1.006 (0.962–1.053)	0.784		
**ER**		0.006		0.873
**Negative**	Reference		Reference	
**Positive**	0.127 (0.029–0.557)		0.722 (0.013–39.536)	
**PR**		0.022		0.542
**Negative**	Reference		Reference	
**Positive**	0.176 (0.040–0.776)		10.814 (0.005–22758.946)	
**HER2**		0.998		
**Negative**	Reference			
**Positive**	0.998 (0.227–4.398)			
**HG**		0.027		0.497
**Ⅰ, Ⅱ**	Reference		Reference	
**Ⅲ**	1.897 (1.077–3.340)		0.625 (0.161–2.429)	
**Tumor size**		0.115		
**≤2 cm**	Reference			
**>2 cm**	2.341 (0.813–6.738)			
**Lymph node metastasis**		0.064		
**Negative**	Reference			
**Positive**	2.542 (0.947–6.825)			
**LVI**		<0.001		0.001
**Negative**	Reference		Reference	
**Positive**	7.406 (2.571–21.334)		6.157 (2.117–17.905)	
**P53 mutation**		0.003		0.044
**Wild type**	Reference		Reference	
**Mutation**	4.751 (1.726–13.073)		2.938 (1.028–8.395)	
**Chemotherapy**		0.087		
**Not done**	Reference			
**Done**	3.653 (0.829–16.105)			
**Radiotherapy**		0.044		0.245
**Not done**	Reference		Reference	
**Done**	4.583 (1.041–20.177)		2.459 (0.540–11.203)	
**Endocrine therapy**		0.003		0.047
**Not done**	Reference		Reference	
**Done**	0.105 (0.024–0.462)		0.211 (0.046–0.978)	

HR, hazard ratio; CIs, confidential intervals; ER, estrogen receptor; PR, progesterone receptor; HER-2, human epidermal growth factor receptor-2; HG, histologic grade; LVI, lymph-vascular invasion.

In the univariate analyses of RFS in low YAP1 tumors, ER, PR, HG, LVI, receipt of endocrine therapy, and mutant p53 pattern were significant factors of poor RFS (Table 3; HR 2.444, 95% CIs 1.448–4.126, *P* = 0.001). Multivariate analysis confirmed mutant p53 pattern was significantly associated with poor RFS in low YAP1 tumors (Table 3; HR 1.842, 95% CIs 1.026–3.304, *P* = 0.041).

**Table 3:** Uni- and multivariate analysis for recurrence-free survival in patients with low nuclear YAP1 expression tumors.

	Univariate analysis		Multivariate analysis	
	HR (95% CIs)	*P* value	HR (95% CIs)	*P* value
**Age (continuous)**	1.005 (0.981–1.029)	0.709		
**ER**		0.003		0.051
**Negative**	Reference		Reference	
**Positive**	0.430 (0.248–0.746)		0.538 (0.288–1.003)	
**PR**		0.009		0.946
**Negative**	Reference		Reference	
**Positive**	0.465 (0.261–0.828)		0.967 (0.366–2.552)	
**HER2**		0.378		
**Negative**	Reference			
**Positive**	0.681 (0.291–1.597)			
**HG**		0.045		0.686
**Ⅰ, Ⅱ**	Reference		Reference	
**Ⅲ**	1.309 (1.006–1.704)		0.869 (0.439–1.719)	
**Tumor size**		0.143		
**≤2 cm**	Reference			
**>2 cm**	1.479 (0.876–2.495)			
**Lymph node metastasis**		0.192		
**Negative**	Reference			
**Positive**	1.415 (0.840–2.384)			
**LVI**		0.033		0.086
**Negative**	Reference		Reference	
**Positive**	1.858 (1.053–3.281)		1.653 (0.931–2.935)	
**P53 mutation**		0.001		0.041
**Wild type**	Reference		Reference	
**Mutation**	2.444 (1.448–4.126)		1.842 (1.026–3.304)	
**Chemotherapy**		0.454		
**Not done**	Reference			
**Done**	1.249 (0.698–2.235)			
**Radiotherapy**		0.445		
**Not done**	Reference			
**Done**	1.233 (0.720–2.113)			
**Endocrine therapy**		0.013		0.453
**Not done**	Reference		Reference	
**Done**	0.515 (0.305–0.869)		1.552 (0.492–4.894)	

HR, hazard ratio; CIs, confidential intervals; ER, estrogen receptor; PR, progesterone receptor; HER-2, human epidermal growth factor receptor-2; HG, histologic grade; LVI, lymph-vascular invasion.

## 4. Discussion

In present study, we identified that mutant p53 pattern was a significant prognostic factor in breast cancer with low nuclear YAP1 expression (OS, HR 6.688, 95% CIs 2.187–20.45, P = 0.0009; RFS, HR 2.771, 95% CIs 1.501–5.115, P = 0.0011), and interestingly, mutant p53 pattern showed no prognostic effect on high YAP1 tumors.

As a downstream effector of the dysregulated Hippo pathway, activation or overexpression of YAP1 contributed to tumor progression in diverse solid tumors including lung cancer [27], bladder cancer [8], ovarian cancer [28], and gastric cancer [29]. However, in breast cancer, the role of YAP1 has been still considered controversial. One study reported the no significant role of YAP1 [30], and the others showed that YAP1 was the favorable predictor, and functioned as a tumor suppressor in breast cancer [31–33]. Conversely, there were also studies that described YAP1 as a poor prognostic factor mediating tumor development and progression of breast cancer [34–36].

Recent study identified that nuclear localization of YAP1 is an activated form of YAP1 [2, 37]. Disruption of the Hippo pathway lost the inhibitory effects on YAP1 and transcriptional coactivator with PDZ-binding motif (TAZ), another Hippo transducer, and results in nuclear retention of YAP1/TAZ. Nuclear-localized YAP1/TAZ binds transcriptional enhanced associate domain (TEAD), and regulates gene transcription related to multiple cancer-associated features [38]. Recent study found that YAP1 activation resulted in fatty acid oxidation and developed lymph node metastasis in mouse model [6].

TP53 is one of the most common mutated gene in many cancers, and studies showed the crosstalk between TP53 and YAP1 [20, 39]. In breast cancer, TP53 mutation has been known to be associated with hormone receptor negativity, high HG, and poor prognosis [40, 41], which is in line with our result. Previous study showed that mutant TP53 and YAP1 shared a common transcriptional signatures, increased expression of cyclin A, cyclin B, cell division cycle 25C, and cyclin-dependent kinase 1 genes [20]. Previous study thus suggested that YAP1 activation could further enhance the effect of mutant TP53, and showed the worst disease-specific survival in mutant TP53 with high YAP1 signature score in METABRIC dataset in breast cancer patient [20]. This study showed sequential survival difference by YAP1 and TP53 status, most favorable survival from wild-type TP53/low YAP1 to wild-type TP53/high YAP1—mutant TP53/low YAP1—mutant TP53/high YAP1. This study had strength in using the molecular data of TP53 and YAP1 status. However, the number of patients was relatively small, and the breast cancers according to molecular subtype were not defined.

In this study, we focused on the effect of YAP1 and p53 expression pattern on breast cancer, with the largest number of breast cancer patient cohort. In present study, case with mutant p53 pattern showed significantly worse OS and RFS among the low YAP1 tumors. Furthermore, mutant p53 pattern was the independent poor prognostic factor in low YAP1 tumors. Intriguingly, there was no statistical difference of OS or RFS depending on the p53 expression pattern in high YAP1 tumors. The RFS graph appeared to be separated, but did not reach the statistical significance. Moreover, the graph was reversed compared to the low YAP1 tumors.

So far, the connection of the Hippo pathway and p53 family proteins has been recognized, but still needs further investigation. In normal state, the Hippo pathway and p53 cooperate as tumor suppressors. Meanwhile, as described above, YAP1 and mutant TP53 could enhance the proliferative effect of each other [20]. Raj et al. deeply discussed the reciprocal crosstalk between Hippo and p53 pathways that interacts in many levels and are closely coordinated [42]. Under wild-type TP53, YAP1 is tightly regulated but loss of TP53 function causes uncontrolled oncogenic activity of YAP1. Also, upstream regulators of both TP53 and YAP1, MDM2 and large tumor suppression 1 (LATS1), could adjust cellular function of PTPN14 that further affects the Hippo pathway signaling. In glioblastoma and breast cancer cells, mutant TP53 establish its oncogenic activity by WASP-interacting protein (WIP), and WIP stabilizes YAP/TAZ thereby sustaining the cancer stem cell survival and oncogenic function [43]. Conversely, loss of LAST 1/2, upstream inhibitor of YAP1, led to conformational change of wild-type TP53, and made similar functional state of mutant TP53 by affecting the TP53 interactome [44]. This change increased YAP1 activity with upregulation of prostaglandin-endoperoxide synthase 2, one of the TP53 target gene, in breast cancer cells [44]. Our non-significant OS and RFS in high YAP1 tumors, those with wild-type p53 pattern might act as functional mutant p53-like state, and that might lead the no differences according to the p53 expression pattern.

Our study has several limitations. First and the most critical limitation is that the p53 expression pattern was determined by immunohistochemistry. In sequencing result, TP53 mutation was not detected in 18.2% of cases with mutant pattern IHC. However, some cases but not all cases that had p53 mutant pattern IHC had mutation pattern on IHC was not consistent with all mutations on TP53 sequencing, all wild0type pattern did not have significant TP53 mutation in sequencing (S1 Table). In addition, there has been studies that correlated the mutation status of TP53 and p53 IHC [26, 45]. However, other confirmative method such as Sanger sequencing would have promised more accurate data and results. One of our result–no difference of survivals of high YAP1 tumors depending p53 expression pattern–also might be originated from this limitation. Second limitation is the small number of high YAP1 tumors. RFS appeared to be separated according to the p53 status in high YAP1 tumors compared to the low YAP1 tumors, although not significant. If the number of high YAP1 tumors were large enough as low YAP1 tumors, survival analysis might have been significantly separated by p53 expression pattern, or showed converted results. As YAP1 and TP53 have reported to interact closely in previous mechanical studies, further study with larger number of high YAP1 tumors is required to verify the association between high YAP1 and mutant TP53. One clinical study with advanced gastric cancer revealed that activated YAP1 along with mutant TP53 status led better survival outcomes, which explained by upregulated proliferative activity of tumor as well as chemosensitivity [46]. As patients in our cohort also treated with standard protocol including appropriate adjuvant chemotherapy, this might explain the trend of superior RFS of mutant p53 pattern and high YAP1 tumors. In addition, TMA slide staining may underestimate the heterogeneity of the tumor compared to whole-slide examination. Despite these limitations, we found relevant evidence showing specific correlations of survival outcomes between YAP1 and p53 expression status.

## 5. Conclusions

In this study, we tried to find the prognostic effect and association of p53 and YAP1 expression pattern in breast cancer. Mutant p53 pattern was strong prognostic factor in low YAP1 tumors but in high YAP1 tumors, that effect seemed to be disappeared. Since real-world clinical data with YAP1 and p53 expression pattern is lacked, further investigation should be performed to evaluate the possible potential of both YAP1 and TP53 as therapeutic targets.

## Supporting information

S1 Table Relationship between p53 expression pattern and mutation sequencing. (DOCX)

## Abbreviations

YAP1: Yes-associated protein 1
TP53: tumor protein 53
HG: histologic grade
ER: estrogen receptor
PR: progesterone receptor
HER2: human epidermal growth receptor-2
LVI: lympho-vascular invasion
IRB: institutional review board
FFPE: formalin-fixed paraffin-embedded
TMA: tissue microarray
IHC: immunohistochemistry
SISH: silver in situ hybridization
OS: overall survival
RFS: recurrence free survival
TNBC: triple negative breast cancer
CI: confidence interval
HR: hazard ratio
TAZ: transcriptional coactivator with PDZ-binding motif
TEAD: transcriptional enhanced associate domain
LAST1: large tumor suppression 1
