# Supplementary material for: The association between the expression of nuclear Yes-associated protein 1 (YAP1) and p53 protein expression profile in breast cancer patients
Source: PLoS One. 2021 May 10;16(5):e0250986. doi: 10.1371/journal.pone.0250986 (PMC8109764; doi:10.1371/journal.pone.0250986)
Supplement: S1 Table — (DOCX) [file pone.0250986.s001.docx]

**S1 Table. Relationship between p53 IHC and mutation sequencing**

|  | **P53 wild type-sequencing** | **P53 mutation-sequencing^a^** |
| --- | --- | --- |
| **P53 wild type-IHC** | 209 (100) | 0 |
| **P53 mutation pattern-IHC** | 12 (18.2%) | 54 (81.8%) |

IHC, immunohistochemistry

^a^Mutation is defined as the case with mutations excluding silent or uncertain significance mutations.
